# A finite strain nonlinear human mitral valve model with fluid-structure interaction

**DOI:** 10.1002/cnm.2691

**Published:** 2014-11-26

**Authors:** Hao Gao, Xingshuang Ma, Nan Qi, Colin Berry, Boyce E Griffith, Xiaoyu Luo

**Affiliations:** 1 School of Mathematics and Statistics, University of GlasgowGlasgow, UK; 2 Bioengineering College, Chongqing UniversityChongqing, China; 3 Institute of Cardiovascular and Medical Sciences, University of GlasgowGlasgow, UK; 4 Department of Mathematics, University of North CarolinaChapel Hill, NC, USA

**Keywords:** human mitral valve, clinical imaging, magnetic resonance imaging, fluid-structure interaction, finite element immersed boundary method, nonlinear finite strain, fibre-reinforced constitutive law

## Abstract

A computational human mitral valve (MV) model under physiological pressure loading is developed using a hybrid finite element immersed boundary method, which incorporates experimentally-based constitutive laws in a three-dimensional fluid-structure interaction framework. A transversely isotropic material constitutive model is used to characterize the mechanical behaviour of the MV tissue based on recent mechanical tests of healthy human mitral leaflets. Our results show good agreement, in terms of the flow rate and the closing and opening configurations, with measurements from in vivo magnetic resonance images. The stresses in the anterior leaflet are found to be higher than those in the posterior leaflet and are concentrated around the annulus trigons and the belly of the leaflet. The results also show that the chordae play an important role in providing a secondary orifice for the flow when the valve opens. Although there are some discrepancies to be overcome in future work, our simulations show that the developed computational model is promising in mimicking the in vivo MV dynamics and providing important information that are not obtainable by in vivo measurements. © 2014 The Authors. *International Journal for Numerical Methods in Biomedical Engineering* published by John Wiley & Sons Ltd.

## 1. INTRODUCTION

Dysfunction of the mitral valve (MV) causes significant morbidity and mortality and remains a major medical problem worldwide. Understanding the biomechanics of human MVs can lead to the development of new therapies and treatment strategies. Changes in MV geometry could perturb the stress patterns and affect repair durability 1,2. However, measuring detailed stress patterns in MVs is extremely challenging in vitro and nearly impossible in vivo. Computational models can fill in the gap by providing estimated stress patterns from structure-based constitutive tissue behaviour, dynamic loading conditions, and in vivo deformation 3–5.

Dynamic modelling of MVs is particularly difficult because of the large deformation of the non-symmetric leaflets, the anisotropic nonlinear elastic behaviour of the valvular tissue, the fluid-structure interaction (FSI), and the pulsatile haemodynamic loading during the cardiac cycles. Biaxial testing of the anterior and posterior leaflets 3,6 has revealed that both leaflets exhibit large deformations and behave anisotropically, being stiffer along the circumferential direction, with the collagen fibres oriented predominantly parallel to the annular ring. Ex vivo mechanical tests on porcine and human mitral apparatus by Prot and colleagues 7–10 and biaxial tests for healthy human mitral leaflets by Wang *et al.* 11 also suggest that the MV leaflets are highly nonlinear and anisotropic.

The in vivo MV mechanical properties are not readily attainable, although limited data have been obtained from in vivo animal tests together with inverse finite element (FE) analysis. Krishnamurthy *et al.* 12 estimated the mechanical response of the anterior mitral leaflets based on radiopaque markers sewn to sheep MVs, albeit with a linear isotropic material model. Lee *et al.* 13 inversely estimated the in vivo material properties on ovine MV anterior leaflets with various nonlinear anisotropic hyperelastic constitutive laws and found that the transversely isotropic law produced the most accurate results.

Computational MV models have been studied by a number of groups, mostly based on structural mechanics approaches. Kunzelman and co-workers were the first to use three-dimensional FE models to simulate normal mitral function 14, the biomechanics of mitral valve disease 15,16, and surgical interventions 16,17. Prot *et al.* 7–9 reported their work on MV simulations in a series of studies using a transversely isotropic strain-energy functional in their nonlinear FE simulations. Their model was later extended to predict the stress distributions between a healthy MV and that in a hypertrophic obstructive cardiomyopathic heart 10. Active muscle contraction of the MV was also considered 18, which is shown to reduce the leaflet bulging significantly from previous FE MV models from this group.

One limitation in most of the aforementioned studies is that the valve geometries were typically taken to be symmetric about the midline of the anterior and posterior leaflets. Three-dimensional dynamic modelling of the ovine MV has been performed by Lim *et al.* 19, who studied the asymmetric stress patterns of the MV. Wenk *et al.* 20 developed an FE model consisting of the left ventricle, mitral apparatus, and chordae tendineae from magnetic resonance (MR) images from a sheep. Recently, Wang *et al.* 11 presented a patient-specific FE model of a healthy human MV reconstructed from multi-slice computed tomography scans with detailed mitral leaflet thickness, chordal information, and mitral annulus dynamic motion. Surgical procedures have also been investigated using structural FE modelling 21–24. The biomechanical response of the valve to the Alfieri stitch technique was reported in 25 and 26. The effects of the annular contraction on MV stress were modelled in 27,28. Other examples of the MV modelling can be found in 4,5,29–32.

Although these structural heart valve models are very useful and suitable for static simulations at the fully opened or fully closed configurations, FSI needs to be accounted for to describe the dynamics of the MV leaflets because of the strong interaction between the blood flow, the leaflets, and the left ventricle 33. Although the arbitrary Lagrangian-Eulerian method is probably the most widely used in FSI simulations, it usually requires dynamic mesh generation for problems that involve large structural deformation and can lead to computational difficulties when dealing with valvular dynamics 34. For this reason, alternative methods, such as immersed boundary (IB) 35,36 and fictitious domain 37,38 methods, have been employed in valvular FSI simulations. Kunzelman, Einstein, and coworkers first started to use a fluid-coupled three-dimensional computational model to simulate normal and pathological mitral function 39–41 and studied the MV closure sound from the closing regurgitation flow. Lau *et al.* 42 investigated the edge-to-edge repair technique with FSI and found that after the repair, the stress is 200% greater than in the normal case, and that there is 44–50% reduction in the peak flow rate. Using a two-dimensional MV model together with left atrium and ventricle, Dahl *et al.* 43 studied MV behaviour during the diastolic filling based on echo data and concluded that the asymmetric leaflet geometry is important for accurately predicting the transvalvular flow patterns. Most of these studies used the commercial package LS-DYNA (Livermore Software Technology Corporation, Livermore, CA) to implement FSI.

We use open source implementations of IB methods for FSI 36,44–46 to simulate MV dynamics. Over the last few years, we have applied the IB method to develop three-dimensional FSI models of a polyurethane prosthetic MV. In a series of studies, we considered the effects of the chordae in the MV 45,46, of the pressure loading condition 35, and of the left ventricular motion 47, which was prescribed using high resolution MRI from a normal human ventricle. We also found that, despite the very thin MV leaflets, their bending rigidity is highly relevant for the effective closing of the MV 48. The bending effect of the MV is also confirmed by a recent study of human MV 44 based on in vivo MRI, which shows that patient-specific MV geometry has a significant influence on the simulation results. One major limitation of these studies is that the MV is modelled with discrete isotropic ‘elastic fibres’, which are not readily able to model the anisotropic hyperelastic mechanical behaviour of the MV leaflets as FE MV models 7,11.

The aim of this study is to overcome the structural simplifications of our previous MV models by developing a combined IB and FE MV model. FE-based structural models for the IB method have been introduced recently 49–53. One of the schemes, a hybrid finite difference-FE IB method (IB/FE) 54, has been carefully verified against commercial package ABAQUS 55, and the results show that the IB/FE model is capable of predicting quantitatively accurate stress/strain distributions for realistic biomechanical models of the heart. In this study, our MV model is again constructed from a human MRI study, but here, the IB/FE approach will enable us to model the MV tissue behaviour using a transversely isotropic model together with the fully dynamic three-dimensional FSI. This is a major step forward from our previous work 44.

## 2. METHODOLOGY

### 2.1 The IB/FE method

The IB/FE approach 54 uses a Lagrangian FE description of the immersed structure along with an Eulerian finite difference description of viscous incompressible fluid, which is modelled using the incompressible Navier-Stokes equations. Let 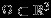 denote the physical domain occupied by the fluid-structure system, and let 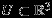 denote the reference coordinate system attached to the MV. Let **x** = (*x*_1_,*x*_2_,*x*_3_)∈Ω denote fixed physical coordinates, let **X** = (*X*_1_,*X*_2_,*X*_3_)∈*U* denote material coordinates attached to the structure, and let ***χ***(**X**,*t*)∈Ω denote the physical position of material point **X** at time *t*, so that ***χ***(*U*,*t*)=Ω_s_(*t*)⊂Ω is the physical region occupied by the structure, and the physical region occupied by the fluid at time *t* is Ω_f_(*t*)=Ω∖Ω_s_. Let **N**(**X**) denote the exterior unit normal to **X**∈*∂U* in the reference configuration. The IB/FE formulation of the equations is

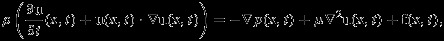
1

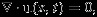
2

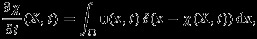
3

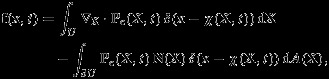
4
in which *ρ* is the mass density, *μ* is the viscosity, **u**(**x**,*t*) is the Eulerian velocity field of the fluid-structure system, *p*(**x**,*t*) is the Eulerian pressure field, **f**(**x**,*t*) is the Eulerian elastic force density, and *δ*(**x**) = *δ*(*x*_1_)*δ*(*x*_2_)*δ*(*x*_3_) is the three-dimensional Dirac delta function. We use the first Piola-Kirchhoff stress tensor 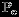 to describe the stresses generated by the immersed structure. 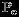 is determined from the passive hyperelastic properties of the leaflets by a strain-energy functional *W* through 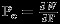, in which 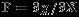 is the deformation gradient associated with the structural deformation. Thus, the total Cauchy stress ***σ*** of the coupled fluid-structure system is

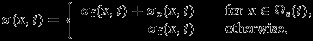
5
***σ***_f_ is the fluid-like stress tensor defined as

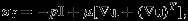
6 in which *p* = *p*(**x**,*t*) is the hydrostatic pressure and ***σ***_e_ is the elastic stress tensor that is related to 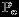 by 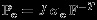, with 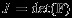


Although we refer to ***σ***_e_ and 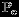 as the structural stress, we remark that they are not the total stresses of the immersed structure but only account for the stresses associated with the hyperelastic material response. The total stress of the immersed structure is 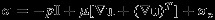; see Eq. (5). Eq. (3) specifies that the velocity of the immersed structure is derived from the Eulerian velocity field, which implies

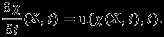
7 Notice that Eq. (7) implies that if ∇·**u** = 0, then *∂J*/*∂t* = 0. Consequently, if *J* = 1 at *t* = *t*_0_, then *J* = 1 for all 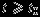.

In the numerical implementation, the Eulerian equations of motion are discretized by a finite difference method, and the Lagrangian equations are discretized with an FE method. Therefore, an equivalent weak formulation of the definition of **f** in (4) is introduced as

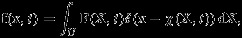
8

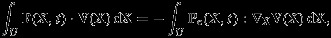
9
in which **F**(**X**,*t*) is the Lagrangian elastic force density and **V**(**X**) is an arbitrary Lagrangian test function that is not assumed to vanish on *∂U*. Although Eqs. (4) and (8)–(9) are equivalent in the continuous setting, they lead to different numerical schemes, and Eqs. (8)–(9) allow **F**(**X**,*t*) to be determined via a standard total Lagrangian FE scheme. Further details are provided by 54.

The simulations described herein employ the open-source IBAMR software framework, which provides an adaptive and distributed-memory parallel implementation of the IB method and an infrastructure for developing FSI models that use the IB method. IBAMR leverages functionality provided by other freely available software libraries, including SAMRAI (https://computation-rnd.llnl.gov/SAMRAI), PETSc (http://www.mcs.anl.gov/petsc) and *hypre* (https://computation-rnd.llnl.gov/linear_solvers).

### 2.2 MV geometrical model

An MRI study was performed on a healthy 28-year-old male volunteer using a 3-Tesla MRI system (Verio, Siemens, Germany). The study was approved by the local NHS Research Ethics Committee, and written informed consent was obtained before the scan. Twelve planes along the left ventricular outflow tract (LVOT) view were imaged to cover the entire MV. Typical parameters are: slice thickness, 3 mm with 0 mm gap; matrix size, 432 × 572; in-plane pixel size, 0.7 × 0.7mm^2^; and frame rate, 25 per cardiac cycle. Short-axis and long-axis views of the ventricle were also acquired to provide additional geometrical information, such as the heads of papillary muscles.

The MV was segmented in the middle of diastole when it is opened by using an in-house MATLAB (The MathWorks Inc., Natick, USA) code. The segmentation method is similar to that in the previous work 44. Detailed steps are described in the Appendix. The MV leaflet are meshed with 154,000 tetrahedral elements with at least two elements across the leaflet thickness.

### 2.3 Material models

The leaflets of the MV are modeled as an incompressible fibre-reinforced material, in which the strain-energy is a functional of invariants of the right Cauchy-Green deformation tensor 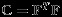 that takes the form

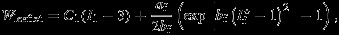
10
in which 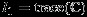 is the first invariant of the right Cauchy-Green deformation tensor and 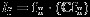 is the square of stretch in the fibre direction, with **f**_0_ denoting the unit tangent to the fibre direction field in the reference state. The modified invariant 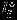 is defined in terms of *I*_f_ by

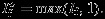
11
This ensures that the load-bearing collagen fibres embedded in the MV leaflets only bear the loads in extension but not compression. The material parameters *C*_1_, *a*_f_, and *b*_f_ were fit to equal-biaxial in vitro tests on a healthy human MV carried out by 11 as shown in Table 1.

**Table 1:** Material parameter values for the strain-energy function *W*_leaflet_.

	*C*_1_ ( kPa)	*a*_f_ (kPa)	*b*_f_
Anterior leaflet	17.4	31.3	55.93
Posterior leaflet	10.2	50.0	63.48

Following our previous study 55, we found that it is useful to employ a modified structural stress tensor 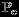 defined as

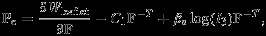
12
in which 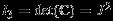 and *β*_*s*_=500 kPa. The pressure-like term 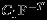 ensures that for 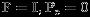, which reduces the magnitude of the pressure discontinuity in the Eulerian pressure field and thereby the magnitude of the spurious volume loss at fluid-solid interface. The term 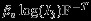 is a penalty term to reinforce the incompressibility constraint in the Lagrangian form, because even though incompressibility is enforced in the Eulerian equations, the numerical interpolation of the Eulerian velocity to the solid region may not always yield a divergence-free discrete Lagrangian velocity field. This additional constraint has been shown to yield more accurate stresses 55.

Although linear tetrahedral elements will yield volumetric locking for sufficiently large values of *β*_*s*_, comparisons to the same MV model with *β*_*s*_=0 verified that for the value of *β*_*s*_ used in this study, our model does not experience volumetric locking because the two models have comparable deformations under the same static loading.

The chordae tendineae are assumed to be isotropic and modelled as a neo-Hookean material:

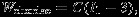
13
in which *C* is spatially homogeneous but takes the value of 9.0 MPa in systole and 0.54 MPa in diastole. These values are based from the measurements of human MV chordae 56. Similarly, the structural tensor 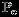 in the chordae tendineae is defined as

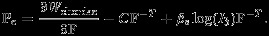
14

### 2.4 Boundary conditions and implementation

In the simulations, we set *ρ* = 1.0g/ml and *μ* = 4cP, and we fix the MV annulus to a housing disc that is mounted in a rigid circular tube immersed in a 10cm × 10cm × 16cm fluid box (Figure 1). The fluid box is discretized with spacings Δ*x*_1_=Δ*x*_2_=0.125cm,Δ*x*_3_=0.1cm, corresponding to a regular 80 × 80 × 160 Cartesian grid. In the numerical scheme, the singular delta function kernel appearing in Eqs. (3) and (8) is replaced by a standard four-point regularized version of the delta function 36. The integral transforms appearing in the interaction equations are approximated using dynamically generated Gaussian quadrature rules that ensure a density of at least two quadrature points per Cartesian mesh width. After computational experimentation, a time step size of 2.5e-5 s is chosen in the explicit time stepping scheme. Further details of the spatial discretizations and time stepping scheme are provided by 54.

**Figure 1:**
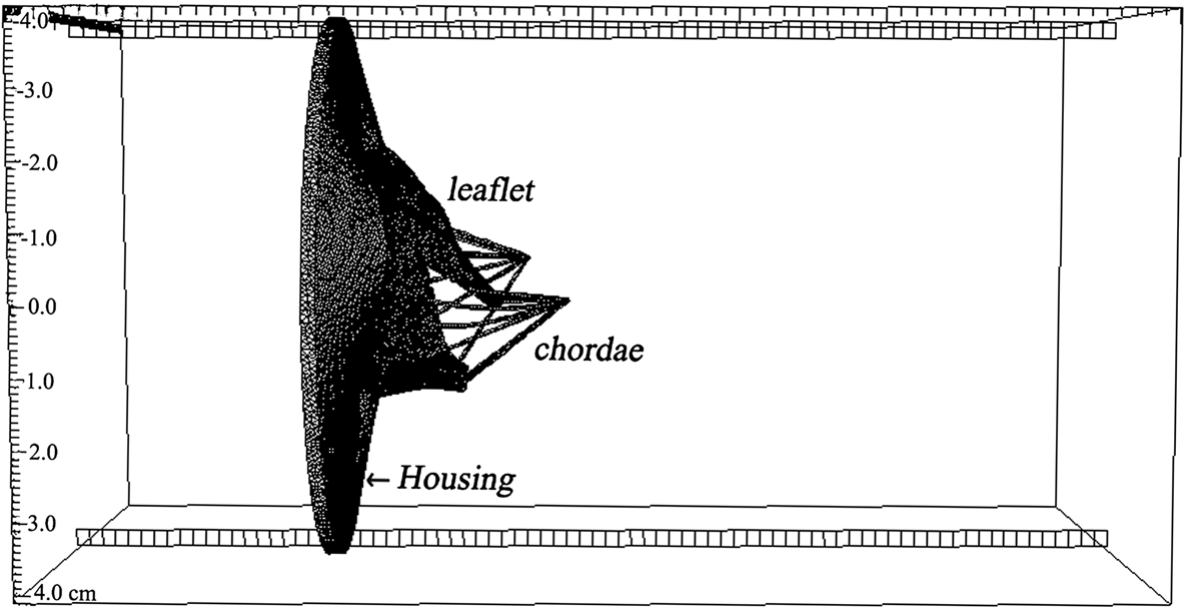
The saddle shaped mitral annulus is fixed to a non-planar rigid housing disc mounted to a semi-rigid circular tube of length 16cm (not shown). The chordae are anchored to two papillary attachment points. These structures are all immersed in a 16cm × 10cm × 10cm rectangular fluid box.

**Figure 2:**
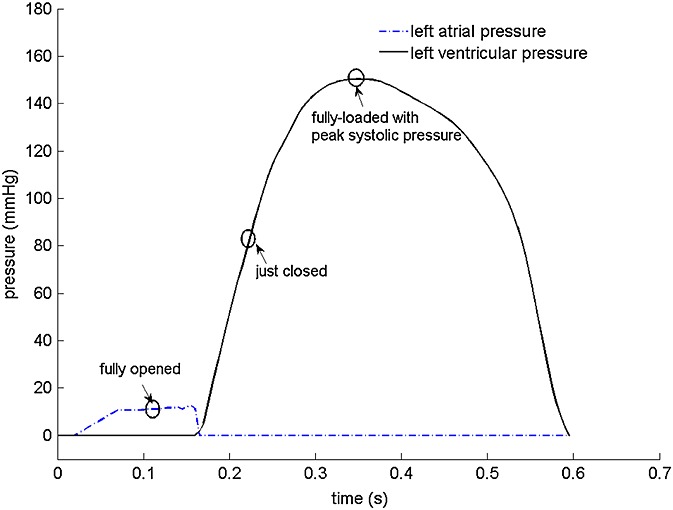
A typical human pressure profile, scaled to the subject-specific peak systolic pressure, is used in the simulations. Note that only the rapid diastolic filling and the systolic phases are modelled; see text for further discussion.

We specify the pressure differences between the inlet and outlet of the tube. Because subject-specific transvalvular pressure data are not available, we instead use a typical human physiological pressure profile rescaled to the cuff pressure of the subject, so that the peak systolic pressure is 150 mmHg §; see Figure 2. We do not model the left ventricle, so only the rapid filling phase of the diastole and the systolic phase are modelled here. This is because in the slow diastolic filling and atrial contraction, the transvalvular flow results mostly from the passive deformation of the left ventricle. Without a model of the left ventricle, slow diastolic filling and atrial contraction cannot be modelled with the proper boundary conditions. This is different from rapid filling, in which the transvalvular flow is due to the suction caused by the left ventricular relaxation. In systole, the boundary conditions can also be easily approximated with the systolic pressure and the papillary muscle movement, as done in other studies 48. The rapid diastolic filling lasts for around 0.2 ms, during which time 80% of transvalvular flow occurs 57. The pressure profile for the part of the cardiac cycle we modelled is shown in Figure 2. Zero-pressure boundary conditions are applied along the remainder of the domain boundaries, similar to our previous study 44.

A grid convergence study is performed with 80 × 80 × 160,96 × 96 × 198, and 112 × 112 × 224 Cartesian grids. Table 2 shows the average and the maximum displacement of the MV model when fully opened and loaded. Notice that only small differences in the computed values are observed for 80 × 80 × 160 when compared with two denser Cartesian grids. For computational efficiency, we use the 80 × 80 × 160 grid for all subsequent simulations.

**Table 2:** Cartesian grid sensitivity analysis, comparing the mean and maximum displacements from the mitral valve leaflets.

	Average displacement (mm)		Maximum displacement (mm)	
Cartesian grid	fully opened	fully loaded	fully opened	fully loaded
80 × 80 × 160	1 ± 1.8	2 ± 3.4	8.2	15.1
96 × 96 × 198	1 ± 1.8	2 ± 3.5	8.3	15.2
112 × 112 × 224	1 ± 1.9	2 ± 3.6	8.6	15.8

Notice that essentially grid-resolved results are obtained for an 80 × 80 × 160 Cartesian grid.

## 3. RESULTS

**Figure 3:**
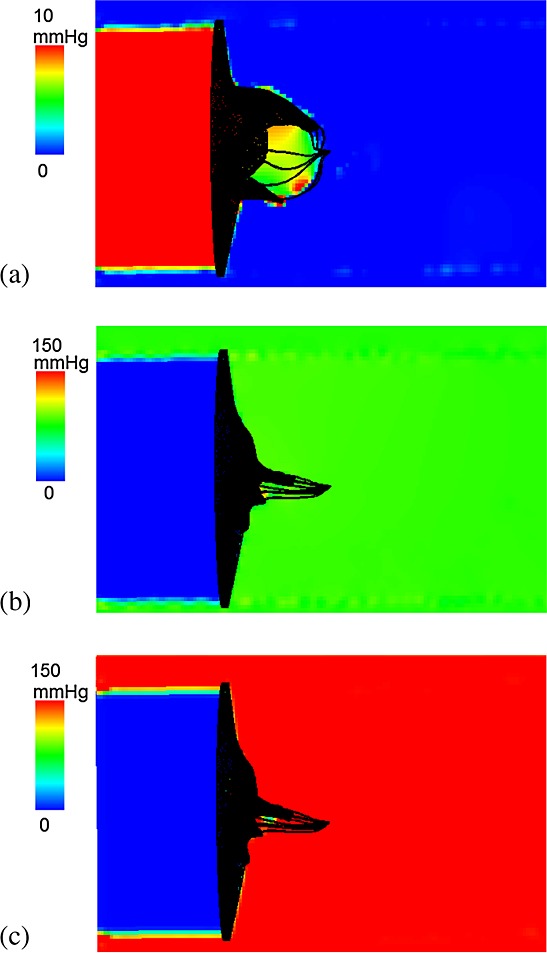
The in-plane view of the fluid pressure field perpendicular to *z*-axis, when the mitral valve is fully opened (a), just closed (b), and fully loaded at the peak pressure of 150 mmHg (c).

The fluid pressure fields from the IB/FE MV model are shown in Figure 3 at the three time instants indicated in Figure 2: when the valve is fully opened (*t* = 0.1 s); just closed (*t* = 0.22 s); and fully loaded at the peak systolic pressure (*t* = 0.35 s). The valve opens at a driving pressure gradient of approximately 10 mmHg and withstands a significant physiological transvalvular pressure gradient when closed. The complete closure of the leaflets occurs at a transvalvular pressure gradient of around 80 mmHg and then undergoes minor further deformation before reaching the peak transvalvular pressure difference (150 mmHg), as shown in Figure 3(b) and (c). Notice that our MV model has small gaps near the commissure area even in the fully closed state, although these are very small and there is no flow leakage in the IB/FE simulations.

**Figure 4:**
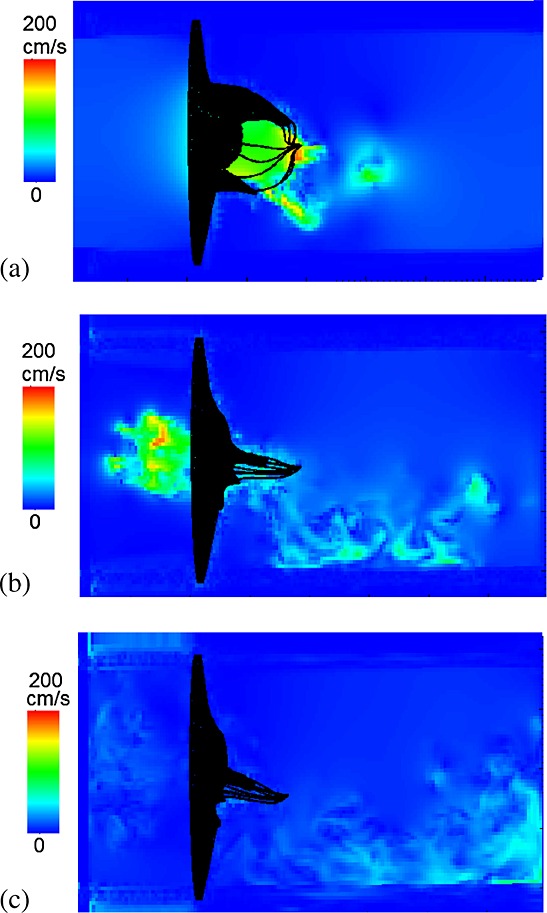
Similar to Figure 3, but here showing the fluid velocity field.

**Figure 5:**
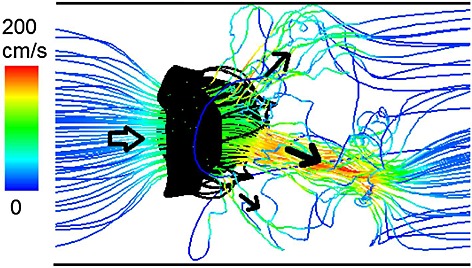
Instantaneous streamlines when the mitral valve is fully open.

The corresponding velocity fields are shown in Figure 4 and indicate a strong jet flow towards the outlet (the left ventricular side) when the MV opens. As the MV closes, there is a closing regurgitation across the MV, as shown in Figure 4(b), which is responsible for the first heart sound. Figure 4(c) shows the velocity pattern when the transvalvular pressure difference peaks. An interesting view is provided by the streamlines when the MV is fully opened, as shown in Figure 5. Here, the flow is first channelled by the two leaflets, then the chordae act as the second orifice 58 and split the jet flow into three paths, with the main stream moving towards the LV apex and side streams flowing towards the walls.

**Figure 6:**
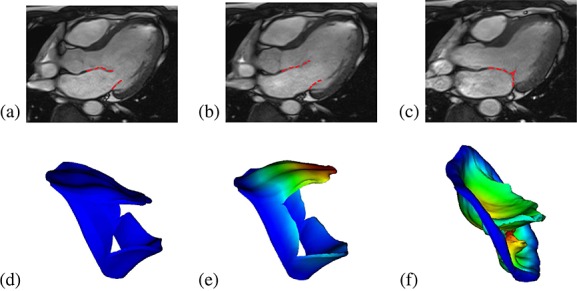
Cine images of the human mitral valve and the sub-valvular apparatus (3-chamber view) showing the MV position at the beginning of the simulation (a), when fully opened (b), and just closed (c). The corresponding MV model predictions are shown in (d, e, f), coloured or greyscaled by displacement magnitude.

The deformed MV leaflets are compared with the corresponding MRI in LVOT view in Figure 6. Qualitatively, the opening and closing configurations of the simulated MV show good agreement with the MRI measurements. However, there is evidence of some discrepancy, particularly in the anterior leaflet when fully closed: the modelled MV bulges into the left atrium, in contrast with that of the corresponding MRI (Figure 6(c)). This is presumably because of simplifications in the chordal model, or the lack of strut chordae.

**Figure 7:**
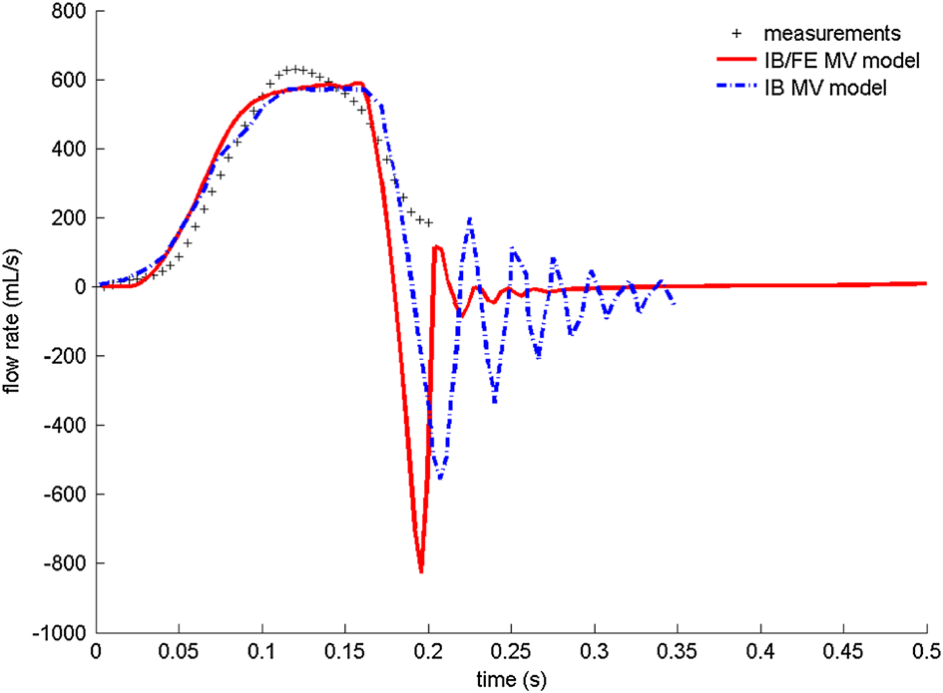
Flow rate comparison between the predictions of the IB/FE model, a previous IB model 44, and the flow measurements obtained from a phase contrast cine magnetic resonance imaging study. Note that only the measured flow rate in rapid filling phase of diastole is plotted, which lasts for 0.2 s.

The flow rate through the valve is shown in Figure 7 along with the measured flow rate from phase contrast MRI, as well as the flow rate obtained from a fibre-based IB MV model 44. When the MV is open, the flow rates from both the IB and IB/FE MV models are comparable with the measured value, with slightly lower peak values. However, the IB/FE model seems to predict a slightly higher regurgitation closing flow (10.8 mL vs. 9.4 mL), followed by much smaller oscillations compared with the previous IB MV model.

**Figure 8:**
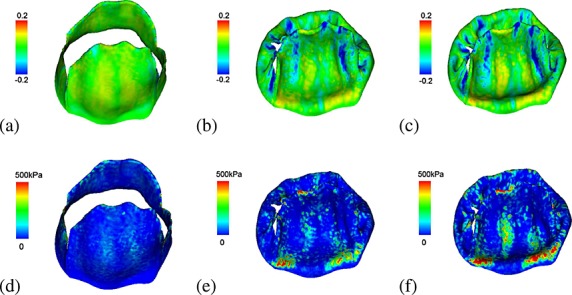
The fibre strain when the mitral valve is fully opened (a), just closed (b), and fully loaded at a transvalvular pressure of 150 mmHg (c). The corresponding fibre stress distributions are shown in (d–f), respectively, which is capped at a peak stress of 500 kPa to remove unrealistic high stress concentrations at the chordae insertion areas.

The stress and strain distributions along the fibre directions are shown in Figure 8. When the MV is fully open, most of the two leaflets are stretched along the fibre direction, and the stress level is lower (Figure 8(a) and (d)). When the MV is just closed, high stress concentrations occur in the fibrous trigones of the anterior leaflet and along the valvular ring. Towards the commissures, there are compressive stresses, particularly in the neighbourhood of the wrinkles. When approaching the highest systolic pressure, the strain and stress patterns (Figure 8(c) and (f)) are similar as the distributions when the MV is just closed (Figure 8(b), (e)), but the high stress area increases, which is most visible in the belly and annular ring regions of the anterior leaflet and higher than the posterior leaflet.

**Figure 9:**
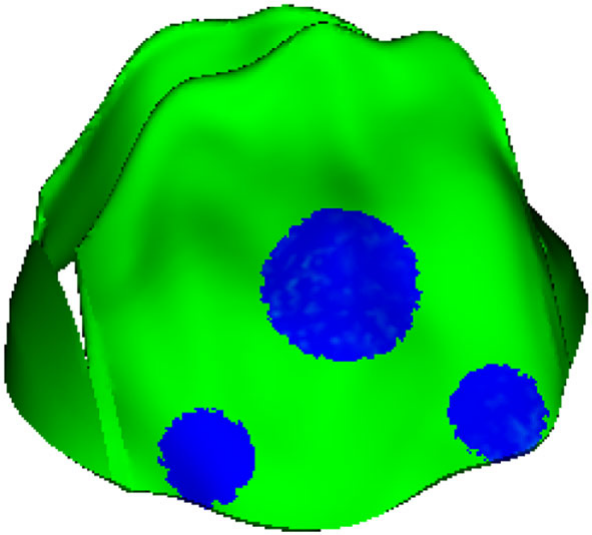
Three local regions in the anterior leaflet are defined for further stress analysis.

We sampled three local regions in the anterior leaflet for detailed analysis of stress and strain. These include two trigones and one belly region, as shown in Figure 9. The average stress and strain of these regions are summarized in Table 3. We can see that when the MV is fully open, the fibres of the belly region are stretched, as indicated by the positive strain, and the stress is higher compared with the trigone regions. Immediately after the MV has closed, the stresses increase in all regions; however, at this point, the two trigones experience higher stress levels compared with the belly region. This pattern remains the same when the MV experiences systolic pressure loads, although the stresses are nearly doubled.

**Table 3:** Average regional stress and strain along the fibre direction on the three local regions defined in Figure 9 when the mitral valve is fully opened, just closed, and fully loaded with peak systolic pressure.

	Stress along fibre directions (kPa)			Strain along fibre directions		
Regions	Fully-opened	Just-closed	Fully-loaded	Fully-opened	Just-closed	Fully-loaded
Trigon 1	−2.9	92	193	−0.004	0.04	0.04
Trigon 2	−0.8	72	144	−0.004	0.02	0.02
The belly region	17	79	142	0.04	0.02	0.02

Table 4 summarizes the average chordae tendineae tension when the MV is fully loaded with the maximum systolic pressure of 150 mmHg. The average force for each chordae associating with the anterior leaflet is 0.68±0.31 N, which is substantially higher than that of the chordae of the posterior leaflet. The tensions in the two papillary muscles are 3.0 N and 3.34 N, respectively. The tension experienced by the chordae and the papillary muscles are comparable with values from other studies 7,60.

**Table 4:** Summary of chordae tendineae tension when the mitral valve is fully loaded with maximum systolic pressure 150 mmHg.

Chordae	Number	Average force (N)
Anterior associated	6	0.68 ± 0.31
Posterior associated	8	0.23 ± 0.15

## 4. DISCUSSION

In this paper, we presented a new IB/FE model for a human MV using in vivo MRI-based anatomy and fibre-reinforced hyperelastic laws. This approach allows us to predict the stress and strain patterns in the MV leaflets with experimentally determined constitutive parameters, which is not possible using our previous IB models 44, as well as to include FSI dynamics. The model is validated by comparing the computed opening and closing configurations and flow rates to clinical measurements from the same volunteer from whom the MV model is constructed.

IB methods have been widely used to simulate the dynamics of the heart and its valves 35,44,48,59,61–63. Most earlier IB MV models used discrete collections of elastic fibres to represent the real MV structure 44. Although these ‘explicit fibre’ models are well suited for describing extremely anisotropic materials, it is challenging to link such descriptions to experimental data and to use hyperelastic constitutive laws designed for fibre-reinforced soft tissue mechanics. Recent extensions of the IB method are able to incorporate finite strain elasticity models for the solid region 49–53, including that used herein. Our results show that this combined IB/FE approach is able to generate a three-dimensional FSI model that can simulate in vivo MV dynamics during diastole and systole.

Compared to our previous work 44, significant improvements are incorporated in the current IB/FE MV model. Namely: (1) the MV leaflets are described with a continuum mechanics framework; (2) the annulus ring is reconstructed from clinical images, not an assumed planar elliptic shape; (3) transversely isotropic constitutive laws are used for the MV leaflets, not the isotropic linear material model; and (4) detailed stress and strain fields are determined, which are not available in our previous IB MV model 44.

Using cine MRI, MV leaflet delineation is most clearly defined in diastole when the transvalvular pressure difference is minimal because the influences from blood flow are minimized 64 and the MV is fully open. By sewing miniature markers onto the MV leaflets of 57 sheep, Rausch *et al.* 65 calculated in vivo strain in the anterior leaflets using reference states corresponding to minimum left ventricular pressure, end-diastole, and the beginning of rapid diastolic filling, and they found that the strain and strain rates are almost insensitive to the choice of the reference configurations. With this in mind, we reconstruct the MV structure in mid-diastole, and because the transvalvular pressure difference is minimal, this is also close to the zero loading state. We also keep the mitral annulus fixed in space, but allow the papillary muscle to move towards the leaflets during systole with a maximum displacement measured from the corresponding cine MRI. Doing this allows the leaflets to close when subjected to a physiological pressure load, as we found previously 46,47.

From Figure 8(a), (b), and (c), it is clear that the strains along the fibre directions are tensile for most of the regions in the anterior leaflet during the cardiac cycle, but some regions that are compressed during closing. Similar results have been reported previously 27,41. Our model prediction of the circumferential strain range seems to agree well with the measurements from in vivo measurements. For example, Rausch *et al.* 65 measured the peak in vivo circumferential strain (along the fibre directions in this study) in the belly region of the anterior leaflet, which is 3.5 ± 3.6*%*. By defining a circular region with a 0.5 cm radius in the belly region of the anterior leaflets, the average strain along fibre directions from our IB/FE model is 3.6 ± 4.0*%*, which is comparable with Rausch's results and also lies in the range reported by Sacks *et al.* 66 from in vivo studies (2.5 − 3.3*%*).

On the other hand, mitral leaflet strain estimated from some in vitro measurements seem to be higher than our predictions. Jimenez *et al.* 67 measured strain in the (porcine) anterior leaflet centre using a left heart simulator, and they found that their peak circumferential strain is 11±4.9% in the normal annular configuration. Similar peak circumferential strain (around 10%) in the leaflet center was also reported in the in vitro studies by Sacks *et al.* 68 and He *et al.* 69,70, although the range of the peak circumferential strain can be as low as 2%  70 (within one standard deviation). The difference in strain between the in vivo and in vitro measurements was also noted by Rausch *et al.* 65. It is not clear if the difference is due to the general difference between the porcine (used in the in vitro studies), ovine (used in the in vivo studies), and human valves (used in our study), or by the incapability of in vitro models to accurately reproduce the mechanical and hemodynamic environment (in terms of the material property, boundary condition and the initial geometry) of the MV. We also note again that the peak systolic pressure of 150 mmHg used in the simulation is slightly higher than a typical value (100–140 mmHg).

The peak average stress along the fibre direction in the belly region is 115 kPa and is 169 kPa and 134 kPa for the two fibrous trigons (Table 3) These predicted stress levels are of similar order to results of other published MV models 5,41. Jimenez *et al.* 60 also measured different types of chordae systolic tension and the forces experienced by the papillary muscles in their in vitro experiments. They reported the peak systolic tension of 0.95±0.35 N in the anterior strut chordae and of 0.35±0.16 N in the anterior marginal chordae. In our model, the average tension in the anterior chordae is 0.68±0.31 N, which is comparable with the range reported by Jimenez 60 and the model results by Wang *et al.* 11. The maximum papillary muscle forces are found to be 4.3 N and 4.6 N from Jimenez's experiments 60, and 4.51 N and 5.17 N from Wang's model 11. These are also comparable with our predications of 3.0 N and 3.34 N.

Although our results are in good agreement with MRI observations and measured flow rates, some model limitations are worth mentioning. For example, the modelled anterior leaflet appears to bulge into the left atrium, in contrast with that of the cine MRI. Also, there are small gaps in the commissure areas when the valve is fully closed. This is due to the difficulties of reconstructing the subvalvular apparatus accurately from cine MRI, especially for the commissure areas and chordae tendineae. We have to build these relying on anatomical descriptions widely adopted in other studies 9,10,30,33,44. More accurate geometry reconstruction may be possible using computed tomography (CT) images. For example, Wang and Sun 11 constructed MV models with chordal origins and insertion points by using multislices CT images. However, CT scans may not be used with healthy human subjects because of the radiation risk. Real time three-dimensional echocardiography could be another promising way to obtain detailed MV structure either non-invasively or with limited invasiveness 30, although the reproducibility is not as good as with CT and MRI. Current in vivo MRI is not able to quantify the fibre directions in the moving MV leaflets, and therefore their distributions are usually modelled using ‘rule-based’ methods 7,30,33,41,44, following experimental observations 6. Recently, Lee *et al.* 71 developed a microanatomical MV models from in vitro experimental measurements by mapping the measured collagen fibre architecture using the small light scattering techniques to the MV models from the same ovine MV leaflets. Non-invasive methods are needed to quantify the in vivo fibre architecture.

Moreover, we have only used simplified primary chordae inserting into the leaflets from the free edges, following our previous work 44–46. In particular, we have not reconstructed the marginal, basal and strut chordae separately, which have different mechanical properties. This may be partially responsible for the MV bulging towards the left atrium. Chordae are bundles of collagen fibres, and some groups modelled these using anisotropic hyperelastic constitutive laws 7,11. We model the chordae as isotropic neo-Hookean material because in diastole, the chordae are usually compressed when MV opens and behave like an isotropic material. In systole, the chordae are stretched and a much higher stress is generated because of the stretched collagen fibres. This is modelled using a much higher chordae stiffness in the systole. Overall, this is a simplified model of the chordae structure that has reproduced the measured in vivo dynamics of MV. With new developments in clinical imaging it may be possible to model more detailed patient-specific chordae structure and its effect on the MV behaviour in the future.

Finally, our initial configuration is partially open, and it is in general very difficult to fully close an opened complex MV configuration that is not initially driven from a fully closed state. Inaccurate geometric details in the commissure areas may be partially responsible; another reason may be that the flows around the MV are not fully physiological due to the absence of the left ventricle.

## 5. CONCLUSION

In this study, we have developed a new human MV model using a hybrid IB/FE approach, which combines FSI simulations with cine MRI and a transversely anisotropic, hyperelastic constitutive model. This model is a significant enhancement from our previous work because it provides dynamic stress distributions, which are found to be concentrated around the annulus trigons and the belly of the anterior leaflet. The model results agree well with the opening and closing leaflet configurations, and with flow rates, estimated from MRI measurements. Although there are still some discrepancies between the model predictions and in vivo observation, with improved imaging techniques and further work, it is possible to develop more realistic MV models that could be applied to study MV diseases.

## Figures and Tables

**Figure:**
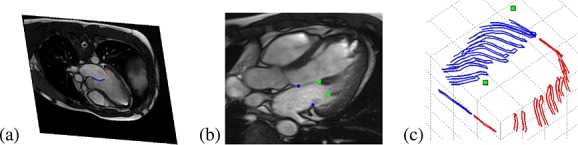
(a) Manual segmentation of leaflets from left ventricular outflow tract images, (b) papillary muscle attachment points shown as the two (green) points on the right, and (c) segmented curves used to assemble the mitral valve leaflets, with the papillary muscle attachment points marked.

**Figure 1:**
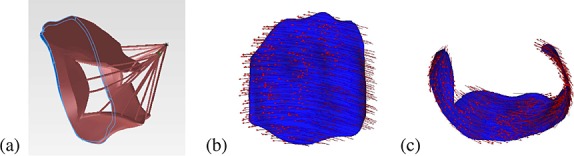
(a) The reconstructed three-dimensional mitral valve model along a spatial annulus ring, with the anterior and posterior leaflets and the chordae. The detailed fibre architecture is shown in (b) for the anterior leaflet and (c) the posterior leaflet.

## APPENDIX

### A. Detailed segmentation of the human MV model

1. *Leaflet segmentation:* the anterior and posterior profiles were manually segmented on cine images of LVOT views (Figure 10(a)). An uniform leaflet thickness of 1 mm was assumed, as shown in Figure 10(c).
2. *Annulus ring:* In each cine image, physical position location that connects the MV leaflets and the ventricular wall were identified as the annulus ring, see Figure 10(b and c), indicated by the blue squares.
3. *Papillary attachment points:* Two papillary muscles were identified from the LVOT and short-axis cine images, as shown in Figure 10(b), indicated by the green squares.
4. *Geometry reconstruction:* The segmented leaflet and papillary heads were imported into SolidWorks (Dassault Systèmes SolidWorks Corp., Waltham, MA, USA) for three-dimensional geometrical reconstruction via B-spline surface fitting, as shown in Figure 11(a).
5. *Chordae tendinae:* A total of 16 evenly distributed primary chordae tendinae were defined based on anatomical descriptions, with 10 associated with the posterior leaflet and six with the anterior leaflet. In our model, the chordae attach to the free margin of the leaflets and run through the leaflets to the annulus ring. These structures model the function of the chordae and we have not separated the contributions from the marginal chordae, strut chordae, and secondary chordae due to the lack of information from the MR images. Similar chordae structure was used in our previous study 44. Each of the chordae is connected to one of the two papillary muscle heads as shown in Figure 11(a), and each is assumed to have a uniform cross-sectional area of 0.16 mm^2^, with a thickness of 0.4 mm and average length of 32 ± 6mm. The papillary heads are allowed to move towards the MV leaflets during systole with displacements measured from the cine MR images, which permits the leaflets to close when subjected to a high systolic pressure.
6. *Collagen fibre architecture:* The embedded collagen fibres were defined along the circumferential direction (parallel to the annulus ring) 3 for both the anterior and the posterior leaflets, as shown in Figure 11(c) and Figure 11(d).

## References

[b1] Bothe W, Kvitting JP, Swanson JC, Hartnett S, Ingels  NB, Miller DC (2010). Effects of different annuloplasty rings on anterior mitral leaflet dimensions. The Journal of Thoracic and Cardiovascular Surgery.

[b2] Bothe W, Kvitting JP, Swanson JC, Göktepe S, Vo KN, Ingels NB, Miller DC (2010). How do annuloplasty rings affect mitral leaflet dynamic motion. European Journal of Cardio-Thoracic Surgery.

[b3] May-Newman K, Yin FCP (1998). A constitutive law for mitral valve tissue. Journal of Biomechanical Engineering.

[b4] Sun W, Martin C, Pham T (2014). Computational modeling of cardiac valve function and intervention. Annual Review of Biomedical Engineering.

[b5] Votta E, Le TB, Stevanella M, Fusini L, Caiani EG, Redaelli A, Sotiropoulos F (2013). Toward patient-specific simulations of cardiac valves: State-of-the-art and future directions. Journal of Biomechanics.

[b6] May-Newman K, Yin FC (1995). Biaxial mechanical behavior of excised porcine mitral valve leaflets. American Journal of Physiology-Heart and Circulatory Physiology.

[b7] Prot V, Haaverstad R, Skallerud B (2009). Finite element analysis of the mitral apparatus: annulus shape effect and chordal force distribution. Biomechanics and Modeling in Mechanobiology.

[b8] Prot V, Skallerud B (2006). An improved transverse isotropic hyperelastic material model for simulation of mitral valve response. Journal of Biomechanics.

[b9] Prot V, Skallerud B (2009). Nonlinear solid finite element analysis of mitral valves with heterogeneous leaflet layers. Computational Mechanics.

[b10] Prot V, Skallerud B, Sommer G, Holzapfel GA (2010). On modelling and analysis of healthy and pathological human mitral valves: Two case studies. Journal of the Mechanical Behavior of Biomedical Materials.

[b11] Wang Q, Sun W (2013). Finite element modelling of mitral valve dynamic deformation using patient-specific multi-slices computed tomography scans. Annals of Biomedical Engineering.

[b12] Krishnamurthy G, Itoh A, Bothe W, Swanson JC, Kuhl E, Karlsson M, Craig Miller D, Ingels Jr NB (2009). Stress–strain behavior of mitral valve leaflets in the beating ovine heart. Journal of Biomechanics.

[b13] Lee CH, Amini R, Gorman RC, Gorman IIIJH, Sacks MS (2013). An inverse modeling approach for stress estimation in mitral valve anterior leaflet valvuloplasty for in-vivo valvular biomaterial assessment. Journal of Biomechanics.

[b14] Kunzelman KS, Cochran RP, Chuong C, Ring WS, Verrier ED, Eberhart RD (1993). Finite element analysis of the mitral valve. Journal of Heart Valve Disease.

[b15] Kunzelman KS, Reimink MS, Cochran RP (1997). Annular dilatation increases stress in the mitral valve and delays coaptation: a finite element computer model. Cardiovascular Surgery.

[b16] Kunzelman KS, Reimink MS, Cochran RP (1998). Flexible versus rigid ring annuloplasty for mitral valve annular dilatation: a finite element model. Journal of Heart Valve Disease.

[b17] Reimink MS, Kunzelman KS, Cochran RP (1996). The effect of chordal replacement suture length on function and stresses in repaired mitral valves: a finite element study. Journal of Heart Valve Disease.

[b18] Skallerud B, Prot V, Nordrum IS (2011). Modeling active muscle contraction in mitral valve leaflets during systole: a first approach. Biomechanics and Modeling in Mechanobiology.

[b19] Lim KH, Yeo JH, Duran CM (2005). Three-dimensional asymmetrical modeling of the mitral valve: a finite element study with dynamic boundaries. Journal of Heart Valve Disease.

[b20] Wenk JF, Zhang Z, Cheng G, Malhotra D, Acevedo-Bolton G, Burger M, Suzuki T, Saloner DA, Wallace AW, Guccione JM, Ratcliffe MB (2010). First finite element model of the left ventricle with mitral valve: insights into ischemic mitral regurgitation. The Annals of Thoracic Surgery.

[b21] Maisano F, Redaelli A, Soncini M, Votta E, Arcobasso L, Alfieri O (2005). An annular prosthesis for the treatment of functional mitral regurgitation: Finite element model analysis of a dog bone–shaped ring prosthesis. The Annals of Thoracic Surgery.

[b22] Urankar SA (2009). Modeling surgical interventions in the mitral valve with the finite element method, University of Pittsburgh.

[b23] Votta E, Maisano F, Alfieri O, Montevecchi FM, Redaelli A (2006). Finite element models of newly shaped prosthetic rings for the correction of functional mitral regurgitation. Journal of Biomechanics.

[b24] Votta E, Maisano F, Bolling SF, Alfieri O, Montevecchi FM, Redaelli A (2007). The geoform disease-specific annuloplasty system: A finite element study. The Annals of Thoracic Surgery.

[b25] Dal Pan F, Donzella G, Fucci C, Schreiber M (2005). Structural effects of an innovative surgical technique to repair heart valve defects. Journal of Biomechanics.

[b26] Votta E, Caiani E, Veronesi F, Soncini M, Montevecchi FM, Redaelli A (2008). Mitral valve finite-element modelling from ultrasound data: a pilot study for a new approach to understand mitral function and clinical scenarios. Philosophical Transactions of the Royal Society A: Mathematical, Physical and Engineering Sciences.

[b27] Stevanella M, Krishnamurthy G, Votta E, Swanson JC, Redaelli A, Ingels Jr NB (2011). Mitral leaflet modeling: Importance of in vivo shape and material roperties. Journal of Biomechanics.

[b28] Stevanella M, Maffessanti F, Conti CA, Votta E, Arnoldi A, Lombardi M, Parodi O, Caiani EG, Redaelli A (2011). Mitral valve patient-specific finite element modeling from cardiac mri: Application to an annuloplasty procedure. Cardiovascular Engineering and Technology.

[b29] Einstein DR, Del Pin F, Jiao X, Kuprat AP, Carson JP, Kunzelman KS, Cochran RP, Guccione JM, Ratcliffe MB (2010). Fluid–structure interactions of the mitral valve and left heart: comprehensive strategies, past, present and future. International Journal for Numerical Methods in Biomedical Engineering.

[b30] Mansi T, Voigt I, Georgescu B, Zheng X, Mengue EA, Hackl M, Ionasec RI, Noack T, Seeburger J, Comaniciu D (2012). An integrated framework for finite-element modeling of mitral valve biomechanics from medical images: Application to mitralclip intervention planning. Medical Image Analysis.

[b31] Sacks MS, David Merryman W, Schmidt DE (2009). On the biomechanics of heart valve function. Journal of Biomechanics.

[b32] Sacks MS, Schoen FJ, Mayer Jr JE (2009). Bioengineering challenges for heart valve tissue engineering. Annual Review of Biomedical Engineering.

[b33] Lau KD, Diaz V, Scambler P, Burriesci G (2010). Mitral valve dynamics in structural and fluid-structure interaction models. Medical Engineering and Physics.

[b34] Van Loon R, Anderson PD, Van de Vosse FN, Sherwin SJ (2007). Comparison of various fluid–structure interaction methods for deformable bodies. Computers and Structures.

[b35] Griffith BE, Luo XY, McQueen DM, Peskin CS (2009). Simulating the fluid dynamics of natural and prosthetic heart valves using the immersed boundary method. International Journal of Applied Mechanics.

[b36] Peskin CS (2002). The immersed boundary method. Acta Numerica.

[b37] De Hart J, Baaijens FPT, Peters GWM, Schreurs PJG (2003). A computational fluid-structure interaction analysis of a fiber-reinforced stentless aortic valve. Journal of Biomechanics.

[b38] van Loon R, Anderson PD, van de Vosse FN (2006). A fluid–structure interaction method with solid-rigid contact for heart valve dynamics. Journal of Computational Physics.

[b39] Einstein DR, Kunzelman KS, Reinhall PG, Nicosia MA, Cochran RP (2005). Non-linear fluid-coupled computational model of the mitral valve. Journal of Heart Valve Disease.

[b40] Einstein DR, Reinhall P, Nicosia M, Cochran RP, Kunzelman K (2003). Dynamic finite element implementation of nonlinear, anisotropic hyperelastic biological membranes. Computer Methods in Biomechanics and Biomedical Engineering.

[b41] Kunzelman KS, Einstein DR, Cochran RP (2007). Fluid–structure interaction models of the mitral valve: function in normal and pathological states. Philosophical Transactions of the Royal Society B: Biological Sciences.

[b42] Lau KD, Díaz-Zuccarini V, Scambler P, Burriesci G (2011). Fluid–structure interaction study of the edge-to-edge repair technique on the mitral valve. Journal of Biomechanics.

[b43] Dahl SK, Vierendeels J, Degroote J, Annerel S, Hellevik LR, Skallerud B (2012). FSI simulation of asymmetric mitral valve dynamics during diastolic filling. Computer Methods in Biomechanics and Biomedical Engineering.

[b44] Ma XS, Gao H, Griffith BE, Berry C, Luo XY (2013). Image-based fluid-structure interaction model of the human mitral valve. Computers and Fluids.

[b45] Watton PN, Luo XY, Wang X, Bernacca GM, Molloy P, Wheatley DJ (2007). Dynamic modelling of prosthetic chorded mitral valves using the immersed boundary method. Journal of Biomechanics.

[b46] Watton PN, Luo XY, Yin M, Bernacca GM, Wheatley DJ (2008). Effect of ventricle motion on the dynamic behaviour of chorded mitral valves. Journal of Fluids and Structures.

[b47] Yin M, Luo XY, Wang TJ, Watton PN (2010). Effects of flow vortex on a chorded mitral valve in the left ventricle. International Journal for Numerical Methods in Biomedical Engineering.

[b48] Luo XY, Griffith BE, Ma XS, Yin M, Wang TJ, Liang CL, Watton PN, Bernacca GM (2012). Effect of bending rigidity in a dynamic model of a polyurethane prosthetic mitral valve. Biomechanics and Modeling in Mechanobiology.

[b49] Boffi D, Gastaldi L, Heltai L, Peskin CS (2008). On the hyper-elastic formulation of the immersed boundary method. Computer Methods in Applied Mechanics and Engineering.

[b50] Heltai L, Costanzo F (2012). Variational implementation of immersed finite element methods. Computer Methods in Applied Mechanics and Engineering.

[b51] Liu WK, Liu YL, Farrell D, Zhang L, Wang XS, Fukui Y, Patankar N, Zhang Y, Bajaj C, Lee J, Hong J, Chen X, Hsu H (2006). Immersed finite element method and its applications to biological systems. Computer Methods in Applied Mechanics and Engineering.

[b52] Zhang L, Gerstenberger A, Wang X, Liu WK (2004). Immersed finite element method. Computer Methods in Applied Mechanics and Engineering.

[b53] Zhang LT, Gay M (2007). Immersed finite element method for fluid-structure interactions. Journal of Fluids and Structures.

[b54] Griffith BE, Luo XY Hybrid finite difference/finite element version of the immersed boundary method.

[b55] Gao H, Wang H, Berry C, Luo XY, Griffith BE (2014). Quasi-static image-based immersed boundary-finite element model of left ventricle under diastolic loading. International Journal for Numerical Methods in Biomedical Engineering.

[b56] Casado JA, Diego S, Ferreño D, Ruiz E, Carrascal I, Méndez D, Revuelta JM, Pontón A, Icardo JM, Gutiérrez-Solana F (2012). Determination of the mechanical properties of normal and calcified human mitral chordae tendineae. Journal of the Mechanical Behavior of Biomedical Materials.

[b57] Nishimura RA, Tajik AJ (1997). Evaluation of diastolic filling of left ventricle in health and disease: Doppler echocardiography is the clinician's rosetta stone. Journal of the American College of Cardiology.

[b58] Davachi F, Moller JH, Edwards JE (1971). Diseases of the mitral valve in infancy an anatomic analysis of 55 cases. Circulation.

[b59] Peskin CS, McQueen DM, Othmer HG, Adler FR, Lewis MA, Dallon JC (1996). Fluid dynamics of the heart and its valves. Case studies in mathematical modeling: Ecology, physiology, and cell biology.

[b60] Jimenez JH, Soerensen DD, He Z, He S, Yoganathan AP (2003). Effects of a saddle shaped annulus on mitral valve function and chordal force distribution: an in vitro study. Annals of Biomedical Engineering.

[b61] Griffith BE (2005). Simulating the blood-muscle-valve mechanics of the heart by an adaptive and parallel version of the immersed boundary method.

[b62] Griffith BE (2012). Immersed boundary model of aortic heart valve dynamics with physiological driving and loading conditions. International Journal of Numerical Methods in Biomedical Engineering.

[b63] Griffith BE, Hornung RD, McQueen DM, Peskin CS (2007). An adaptive, formally second order accurate version of the immersed boundary method. Journal of Computational Physics.

[b64] Ferreira PF, Gatehouse PD, Mohiaddin RH, Firmin DN (2013). Cardiovascular magnetic resonance artefacts. Journal of Cardiovascular Magnetic Resonance.

[b65] Rausch MK, Bothe W, Kvitting JPE, Göktepe S, Craig Miller D, Kuhl E (2011). In vivo dynamic strains of the ovine anterior mitral valve leaflet. Journal of Biomechanics.

[b66] Sacks MS, Enomoto Y, Graybill JR, Merryman WD, Zeeshan A, Yoganathan AP, Levy RJ, Gorman RC, Gorman III JH (2006). In-vivo dynamic deformation of the mitral valve anterior leaflet. The Annals of Thoracic Surgery.

[b67] Jimenez H, Liou SW, Padala M, He Z, Sacks M, Gorman RC, Gorman III JH, Yoganathan AP (2007). A saddle-shaped annulus reduces systolic strain on the central region of the mitral valve anterior leaflet. The Journal of Thoracic and Cardiovascular Surgery.

[b68] Sacks MS, He Z, Baijens L, Wanant S, Shah P, Sugimoto H, Yoganathan AP (2002). Surface strains in the anterior leaflet of the functioning mitral valve. Annals of Biomedical Engineering.

[b69] He Z, Ritchie J, Grashow JS, Sacks MS, Yoganathan AP (2005). In vitro dynamic strain behavior of the mitral valve posterior leaflet. Journal of Biomechanical Engineering.

[b70] He Z, Sacks MS, Baijens L, Wanant S, Shah P, Yoganathan AP (2003). Effects of papillary muscle position on in-vitro dynamic strain on the porcine mitral valve. The Journal of Heart Valve Disease.

[b71] Lee CH, Oomen PJA, Rabbah JP, Yoganathan A, Gorman RC, Gorman III JH, Amini R, Sacks MS (2013). A high-fidelity and micro-anatomically accurate 3D finite element model for simulations of functional mitral valve. Functional Imaging and Modeling of the Heart.

